# Elevated ZC3H15 increases HCC growth and predicts poor survival after surgical resection

**DOI:** 10.18632/oncotarget.9361

**Published:** 2016-05-14

**Authors:** Bei-ge Jiang, Zheng-hua Wan, Jian Huang, Li-mei Li, Hui Liu, Si-yuan Fu, Yuan Yang, Jin Zhang, Shen-xian Yuan, Ruo-yu Wang, Yun Yang, Fang-ming Gu, Li-wei Dong, Ze-ya Pan, Wei-ping Zhou

**Affiliations:** ^1^ Hepatic Surgery Department III, Eastern Hepatobiliary Surgery Hospital, The Second Military Medical University, National Innovation Alliance for Hepatitis & Liver Cancer, Shanghai, P. R. China; ^2^ International Cooperation Laboratory on Signal Transduction, Eastern Hepatobiliary Surgery Institute, The Second Military Medical University, Shanghai, P. R. China; ^3^ State Key Laboratory of Cell Biology, Institute of Biochemistry and Cell biology, Shanghai Institutes for Biological Sciences, Chinese Academy of Sciences, Shanghai, P. R. China

**Keywords:** hepatocellular carcinoma, ZC3H15, NFκB, TRAF2

## Abstract

Zinc finger CCCH-type containing 15 (ZC3H15), also known as DRG family regulatory protein 1 (DFRP1), is a highly conserved eukaryotic protein that associates with active translation machinery. The aim of our study was to explore the clinical relevance and intrinsic functions of ZC3H15 in hepatocellular carcinoma (HCC). We constructed a cohort with 261 tumor and matched normal tissues from HCC patients. ZC3H15 protein and mRNA levels were determined using immunohistochemistry, western blot analysis, and quantitative polymerase chain reaction. ZC3H15 was highly expressed in the majority of HCC cases, and high ZC3H15 levels were significantly associated with high serum a-fetoprotein (AFP) levels (>20 ng/mL) and vascular invasion. Kaplan-Meier and Cox regression data indicated that elevated ZC3H15 was an independent predictor for HCC-specific disease-free survival (hazards ratio [HR], 1.789; 95% confidence interval [95% CI], 1.298-2.466 [P=0.0004]) and overall survival (HR, 1.613; 95% CI, 1.120-2.322 [P=0.0101]). Interaction of ZC3H15 with TRAF2 increased activation of NFκB signaling. These results suggest ZC3H15 is an independent prognostic marker in HCC patients that is clinicopathologically associated with tumor invasion and serum AFP levels.

## INTRODUCTION

Hepatocellular carcinoma (HCC) is among the most lethal and aggressive neoplasms [1] and the second leading cause of cancer death in China. Hepatitis B virus (HBV) is the most significant risk factor for HCC [2–4]. HCCs that grow rapidly with early vascular invasion are highly resistant to chemotherapy [5–7], and the high frequency of tumor recurrence or distant metastasis after surgical resection leads to extremely poor prognosis for patients with HCC [8].

The developmentally regulated GTP-binding (DRG) protein subfamily is a branch of the GTPase superfamily with members found throughout the eukaryotes and archaea [9]. The DRG orthologs DRG1 and DRG2 are similar to two paralogs found in most eukaryotic genomes [10, 11]. For example, they share 57% identity with the two human paralogs and 62% identity with the Saccharomyces cerevisiae paralogs [12]. Mammalian DRG1 and DRG2 interact with DFRP1 (ZC3H15) and DFRP2, respectively [13]. ZC3H15 and DFRP2 proteins contain a conserved DRG family regulatory protein (DFRP) domain (~60 aa) identified using multiple alignment of sequences from mouse, fly, and yeast. Both ZC3H15 and DFRP2 are highly conserved in eukaryotes such that there is respectively 51% and 46% similarity between the human and the yeast orthologs [13]. The DFRP domain is required for association of DFRP1/2 with DRGs [13]. ZC3H15 also contains a characteristic tandem repeat CCCH zinc finger domain (TZF) with significant similarity to RNA-binding proteins [14], suggesting ZC3H15 function is linked to RNA metabolism. Moreover, recent observations suggest the yeast orthologs of DRG1, ZC3H15, and DFRP2 physically associate with active translation machinery [15, 16].

The purpose of this study is to investigate the expression of ZC3H15 and its association with liver malignancy and to determine its prognostic value for clinical outcomes. Using an expression profile microarray, we found that ZC3H15 expression is higher in HCC than adjacent normal liver tissue, whereas DFRP2 expression did not differ between the two tissue types.

## RESULTS

### ZC3H15 is overexpressed in HCC specimens

**Figure 1:**
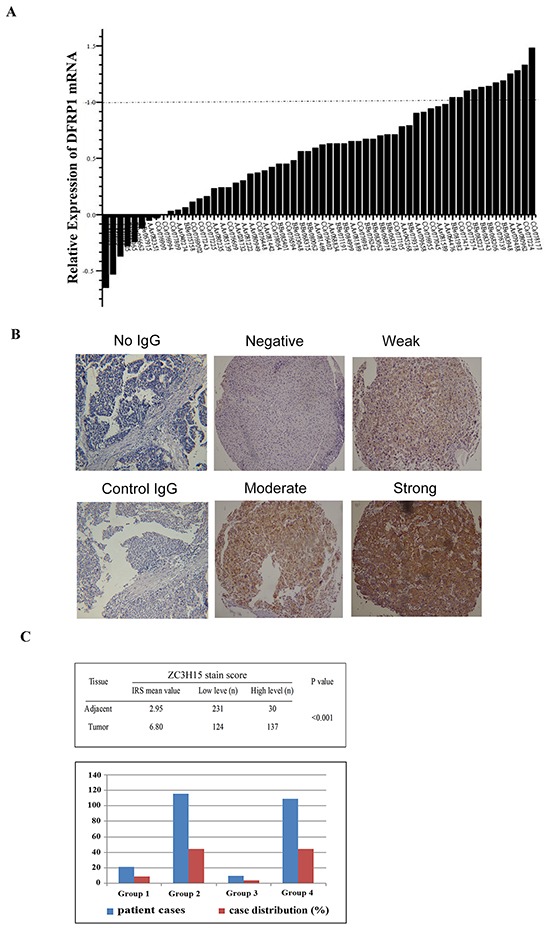
ZC3H15 (DFRP1) is highly expressed in HCC specimens **A.** ZC3H15 mRNA expression in HCC and adjacent normal-like liver tissues. The value represents relative level of each HCC tumor to the peritumoral normal tissue mRNA. **B.** Immunohistochemistry analysis of ZC3H15 protein levels in HCC and normal liver tissues. **C.** Distribution of patients according to ZC3H15 immunofluorescence. Based on ZC3H15 IRS scores, patients divided into high and low expression groups (Table). Graphical representation of patients’ distribution according to ZC3H15 expression levels. Group1: tumor high and adjacent normal tissue high, Group2: tumor high and adjacent normal tissue low, Group3: tumor low and adjacent normal tissue high, Group3: both low.

We determined the ZC3H15 levels by quantitative RT-PCR and immunohistochemistry (IHC) in a retrospective cohort of tumor tissues and matched normal tissues samples from HCC patients after liver resection. ZC3H15 mRNA levels were examined in 60 pairs of tumor and normal liver tissues. 52 out of 60 cases displayed higher ZC3H15 transcripts in tumor than that in adjacent non-tumor tissues (Figure 1A). IHC staining intensity for ZC3H15 was much stronger in tumor than in adjacent normal-like tissues and normal tissues, and the ZC3H15 score was significantly higher in tumor cells than in non-tumor counterparts and normal specimens (p<0.001; Figure 1B, 1C). ZC3H15 predominantly presented in the cytoplasm of tumor cells, and only a few tumor cells showed positive ZC3H15 staining in the nuclei (Figure 1B). In the 276 cases examined, 137 specimens (52.5%) and 30 non-tumor specimens (11.5%) had a high level of ZC3H15. According to ZC3H15 IRS scores, patients were divided into four groups and in most tissues ZC3H15 was highly expressed in tumor and low in peritumoral counterpart tissues (Figure 1C). ZC3H15 protein and mRNA levels were approximately 2 to 3 times higher in tumors than in matched non-tumor tissues.

### Association of ZC3H15 expression with clinicopathological features

Next, clinical association analysis by the Pearson chi-square test revealed that ZC3H15 level in HCC tumors was significantly associated with high serum AFP levels (>20 ng/mL; p < 0.0064) and vascular invasion (p<0.0049; Table 1). Other clinical characteristics, including age, gender, tumor size, distant metastasis, encapsulation, UICC stage, and hepatitis background were not directly related to the expression of ZC3H15. To validate the result, small HCC (tumor size < 3cm) was selected for analysis of correlation of ZC3H15 expression with the clinicopathological features. Only serum AFP levels were associated with ZC3H15 protein levels (p<0.058; Supplementary Table S1).

**Table 1:** Relationship between ZC3H15 protein expression and clinicopathologic characteristics(n=261*)

Characteristics	No. patients(%)	ZC3H15 immunoreactivity*		P value
		High	Low	
Gender				
Male	230(88.12)	125	105	0.1017
Female	31(11.88)	12	19	
Age (yrs)				
<40	38(14.55)	20	18	0.6719
40-60	166(63.60)	90	76	
>60	57(21.84)	27	30	
AFP				
≤20 (ng/mL)	98(37.69)	41	57	**0.0064**
>20	163(62.31)	97	66	
HBV				
Negative	33(12.64)	14	19	0.2154
Positive	228(87.36)	123	105	
Histological grading				
Well	1(0.36)	0	1	0.0705
Moderate	25(10.51)	8	17	
Poorly	234(88.77)	129	105	
Largest tumor size				
<3cm	70(26.82)	39	31	0.6355
3-10cm	152(58.24)	76	76	
>10cm	39(14.94)	22	17	
Presence of satellite nodules				
Negative	73(27.97)	33	40	0.1419
Positive	188(72.03)	104	84	
Vascular invasion				
Negative	109(41.76)	46	63	**0.0049**
Positive	152(58.23)	91	61	
HbeAg				
Negative	202(77.39)	102	100	0.2323
Positive	59(22.61)	35	24	
Tumorous number				
1	208(79.69)	108	100	0.7162
>=2	53(20.30)	29	24	
Child-Pugh Classification				
Child-A	237(90.80)	123	114	0.5542
Child-B	24(9.20)	11	13	
UICC stage				
I+II	204(78.16)	106	98	0.7458
III+IV	57(21.84)	31	26	

* For ZC3H15, median values were used as the cutoff point for definition of subgroups (low expression and high expression groups)

### Prognostic value of ZC3H15 expression

**Figure 2:**
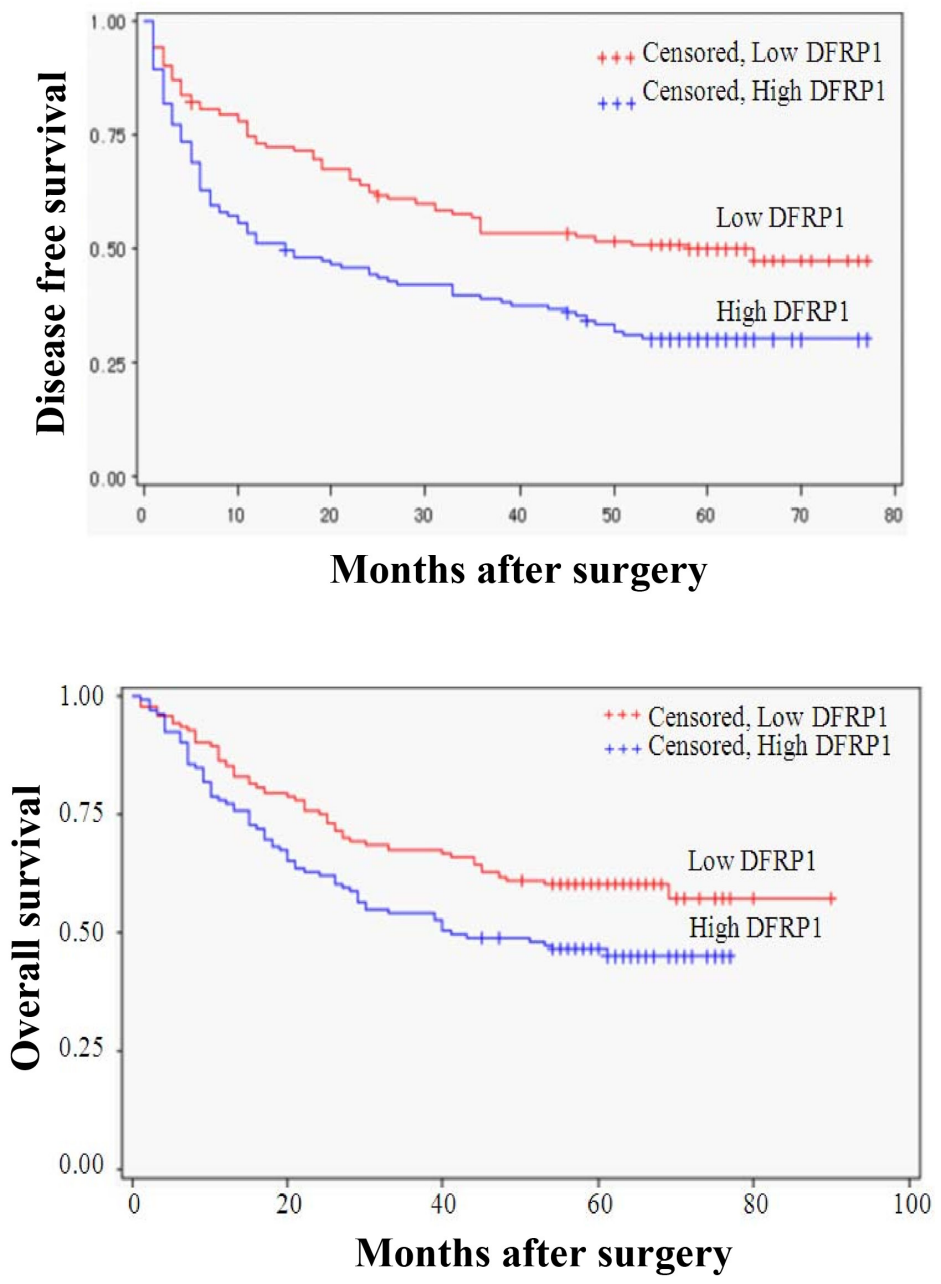
Kaplan-Meier curves for time to recurrence and overall survival of patients with high or low intratumoral ZC3H15 (DFRP1) features The overall survival rate (OS) and recurrence rate were analyzed in ZC3H15 low and high group. Subgroups were plotted according to the scores of ZC3H15 levels.

The overall survival rate (OS) and recurrence rate were 51.09% and 61.96%, respectively, for all patients in this study. Kaplan–Meier analysis revealed that the high-ZC3H15 patients had much shorter overall survival and higher recurrence rates than the low-ZC3H15 patients (Figure 2). Univariate analysis of clinical variables considered as potential predictors of survival is shown in Table 2. Univariate analysis revealed that ZC3H15, serum AFP, HBV, tumor size, venous invasion, and UICC stages could serve as predictors for OS or/and DFS. Multivariate Cox regression indicated that ZC3H15, together with tumor size, HBV, and UICC stage were strongly associated with OS. ZC3H15 also correlated with DFS in our study cohort. Thus, ZC3H15 was an independent prognostic indicator for OS (p=0.0101) and DFS (p=0.0004).

**Table 2:** Univariate and multivariate Cox regression analyses ZC3H15 for DFS or OS of patients(n=261)

Variables	DFS		OS	
	Hazard ratio (95% CI)*	p Value	Hazard ratio (95% CI)*	p Value
**Univariate analysis**				
ZC3H15(low vs high)	1.789(1.301-2.460)	**0.0003**	1.588(1.114-2.264)	**0.0105**
Gender (male vs female)	0.950(0.583-1.549)	0.8377	1.167(0.692-1.966)	0.5624
Age, years (<40 vs 40-60 vs >60)	1.065(0.834-1.359)	0.6156	0.989(0.751-1.304)	0.94
Child-Pugh Classification(A vs B)	1.580(0.978-2.551)	0.0614	1.675(0.992-2.830)	0.0538
Serum AFP, ng/ml (>20 vs≤20)	1.405(1.025-1.927)	**0.0348**	1.262(0.887-1.795)	0.1959
HBV (negative vs positive)	2.347(1.332-4.136)	**0.0032**	2.113(1.109-4.026)	**0.0229**
Histological grading(well vs moderate vs poorly)	1.397(0.875-2.230)	0.1616	1.219(0.726-2.046)	0.4548
Largest tumor size, cm(<3 vs 3-10 vs >10)	1.664(1.311-2.112)	**<0.0001**	1.957(1.488-2.573)	**<0.0001**
Presence of satellite nodules (negative vs positive)	1.212(0.860-1.708)	0.2719	1.043(0.717-1.518)	0.8254
Vascular invasion(negative vs positive)	1.669(1.221-2.282)	**0.0013**	1.712(1.201-2.442)	**0.003**
HbeAg(negative vs positive)	1.691(1.207-2.369)	**0.0022**	1.523(1.046-2.218)	**0.0283**
Tumorous number	1.334(0.927-1.920)	0.1212	1.511(1.020-2.238)	**0.0394**
UICC stage (I+II vs III+IV)	1.943(1.380-2.737)	**0.0001**	2.310(1.599-3.339)	**<0.0001**
**Multivariate analysis**				
ZC3H15(low vs high)	1.789(1.298-2.466)	**0.0004**	1.613(1.120-2.322)	**0.0101**
Gender (male vs female)	NA			
Age, years (<40 vs 40-60 vs >60)	NA			
Child-Pugh Classification(A vs B)	NA		2.038(1.182-3.513)	0.0104*
Serum AFP, ng/ml (>20 vs≤20)	NA			
HBV (negative vs positive)	2.494(1.378-4.517)	**0.0025**	2.433(1.227-4.822)	**0.0109**
Histological grading(well vs moderate vs poorly)	NA			
Largest tumor size, cm(<3 vs 3-10 vs >10)	1.866(1.442-2.415)	**<0.0001**	2.253(1.661-3.055)	**<0.0001**
Presence of satellite nodules (negative vs positive)	NA			
Vascular invasion(negative vs positive)	NA			
HbeAg(negative vs positive)	1.641(1.161-2.319)	**0.005**		
Tumorous number	NA			
UICC stage (I+II vs III+IV)	1.751(1.224-2.503)	**0.0021**	2.365(1.601-3.492)	**<0.0001**

* For ZC3H15, median values were used as the cut-off point for definition of subgroups (low expression and high expression groups).

Univariate analysis, Cox proportional hazards regression; Multivariate analysis, Cox proportional hazards regression; Variables were adopted in multivariate analysis for their prognostic significance by univariate analysis.

### ZC3H15 knockdown inhibited cell proliferation

**Figure 3:**
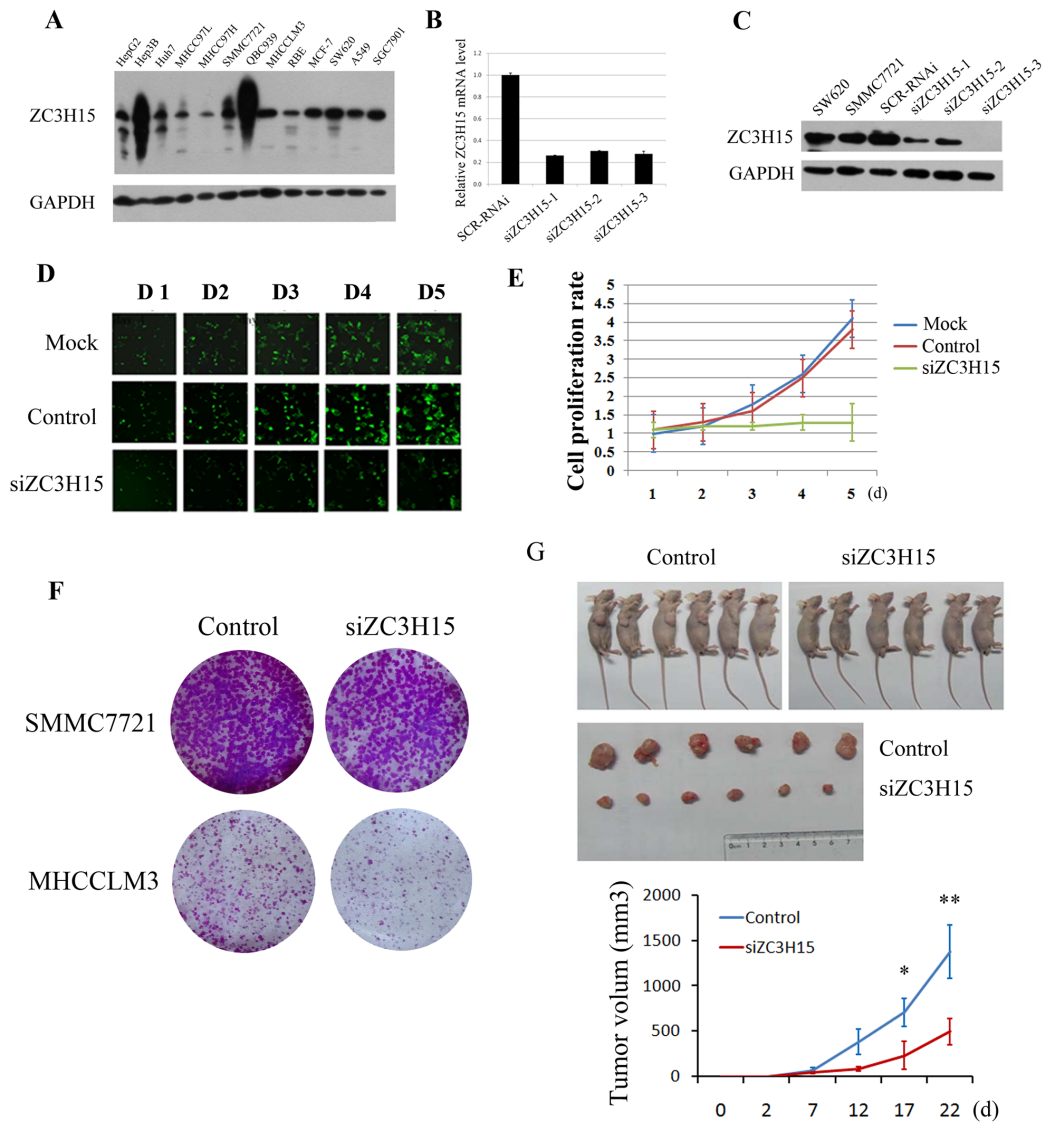
ZC3H15 knockdown inhibited HCC cell growth *in vitro* and *in vivo* **A.** ZC3H15 protein levels were examined by western blotting in HCC and other cell lines. **B, C.** ZC3H15 was knocked down in SMMC-7721 cells by lentivirus delivered shRNA targeting ZC3H15. The effects of the three shRNA were examined by qPCR and western blotting assays. **D, E.** Proliferation of siZC3H15-SMMC-7721 and control cells was detected by quantifying EGFP fluorescence intensity. **F.** A panel clonogenic assay of two HCC cell lines after ZC3H15 knockdown. The HCC cells (2×10^3^ cells per well) were seeded in 6-well plates and incubated for 10 days, and then the number of cell colonies was counted. **G.** SMMC7721-control and siZC3H15 cells (5×10^6^ cells per mouse) were inoculated into flanks of nude mice and tumor growth was observed for 3 weeks. Error bars represent the SEM *P<0.05, **P<0.01.

ZC3H15 protein and mRNA were quantified in HCC and other cell lines (Figure 3A). ZC3H15 is widely expressed in various cell lines including HCC. ZC3H15 mRNA and protein levels were reduced in SMMC7721 cells through lentivirus infection (Figure 3B, 3C). We examined proliferation of these cells by measuring EGFP fluorescence intensity up to five days and found that knockdown of ZC3H15 inhibited cell proliferation (Figure 3D, 3E). Furthermore, ZC3H15 knockdown resulted in decreased colony formation for both SMMC7721 and MHCCLMS cells compared with control cells (Figure 3F). SMMC7721-siZC3H15 and control cells were inoculated into flanks of nude mice. Knockdown of ZC3H15 significantly decreased tumor size (Figure 3G).

### ZC3H15 inhibited HCC apoptosis by activating NFκB signaling

**Figure 4:**
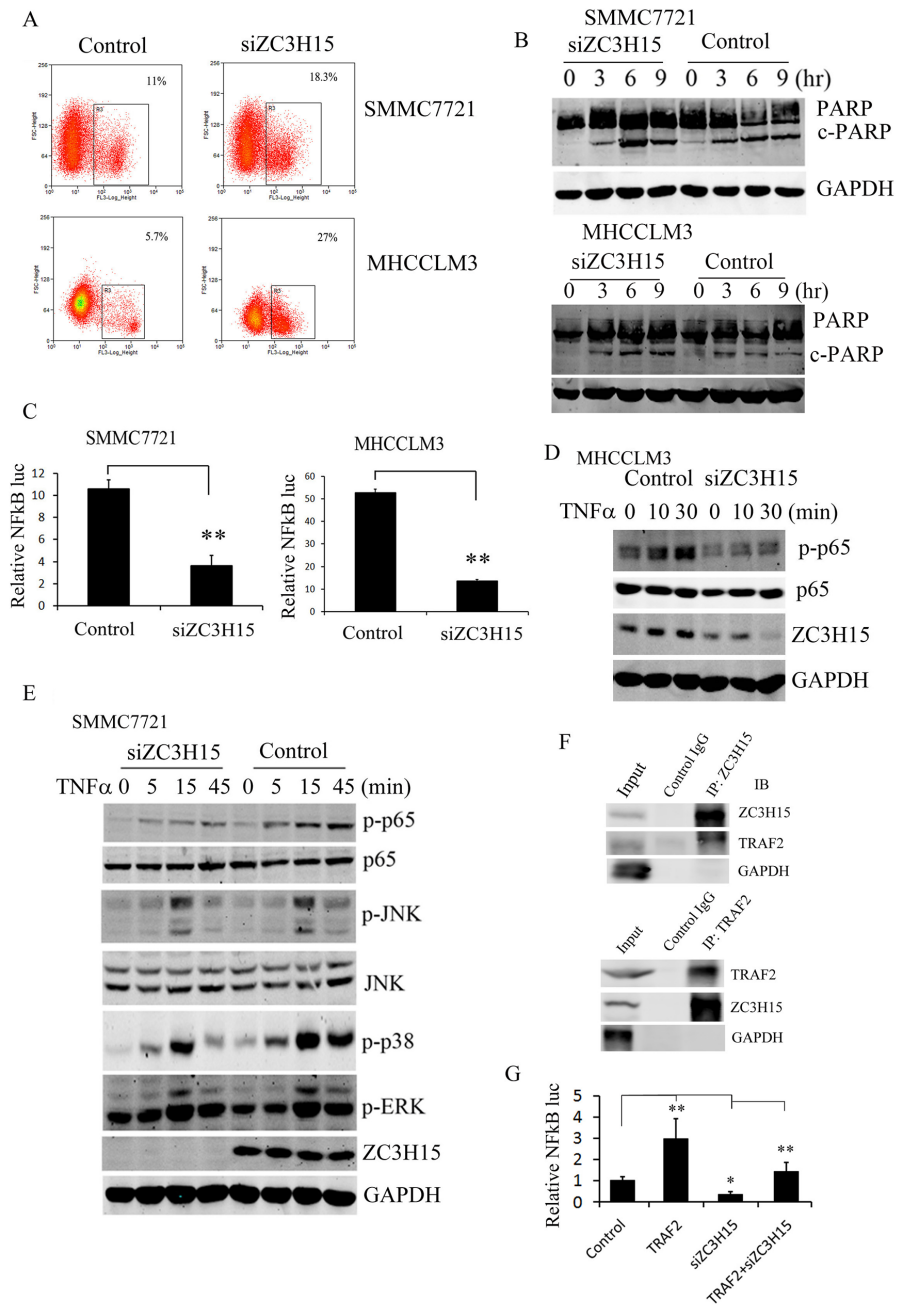
ZC3H15 inhibited apoptosis of HCC by activating NFκB signaling **A.** ZC3H15 knockdown induced HCC cell apoptosis. SMMC7721 and MHCCLM3 cells were infected with lentivirus-delivered siZC3H15. After 3 days, cell apoptosis was measured by flow cytometry. **B.** Western blot analysis to examine cleaved PARP level in SMMC7721-control and -siZC3H15 cells. **C.** Inhibition of ZC3H15 expression attenuated activation of NFκB. SMMC7721 or MHCCLM3-control and -siZC3H15 cells were co-transfected with a mixture of luciferase reporter plasmid and pRL-TK plasmids. After 24 h, luciferase activities were measured. **D, E.** TNFα induced p-p65 and MAPKs activation were suppressed by knockdown ZC3H15. SMMC7721 or MHCCLM3-control and -siZC3H15 cells were treated with TNFα for 0 to 45 minutes and then lysed for western blot analysis. **F.** SMMC7721 cells were lysed and immunoprecipitated with anti-ZC3H15 or anti-TRAF2 antibody. Precipitates and cell lysates were blotted with indicated antibodies. **G.** TRAF2 expression rescued the decrease of the transcriptional activity caused by the loss of ZC3H15. SMMC7721-control and -siZC3H15 cells were transfected with TRAF2 vectors for 36 hours and NFκB luciferase activity was measured.

Flow cytometry assay revealed that suppression of ZC3H15 induced apoptosis in a large number of cells (Figure 4A). Cleaved PARP was also detected in SMMC7721 cells upon ZC3H15 depletion (Figure 4B). ZC3H15 knockdown significantly increased the levels of cleaved PARP when HCC cells were subjected to TNFα plus CHX treatment. When apoptosis was triggered by TNFα, a paralleled pathway, NFκB signaling, was activated by its stimulation, which can help cells resist apoptosis. NFκB luciferase reporter activity was detected in SMMC7721 and MHCCLM3 cells with or without ZC3H15 inhibition. As shown in Figure 4C, NFκB transcriptional activity was attenuated by ZC3H15 silencing. Further, phosphorylation of NFκB subunit p65 was examined in MHCCLM3 or SMMC7721-control and -siZC3H15 cells (Figure 4D, 4E). Western blot revealed that TNFα stimulation gave rise to much higher p-p65 levels in control cells than in siZC3H15 cells, indicating ZC3H15 activation of NFκB. TNFα-dependent activation of MAPK pathways was detected in SMMC7721-control and -siZC3H15 cells. Phosphorylated JNK, p38, and ERK were all decreased by ZC3H15 knockdown (Figure 4E). Because Previous ZC3H15 physically associates with TRAF2, the interaction of ZC3H15 with human TRAF2 was studied by co-immunoprecipitation (Figure 4F). The ZC3H15 or TRAF2-specific antibody was used to precipitate their associated protein, respectively. The subsequent western blot analysis of the precipitates revealed that TRAF2 was associated with ZC3H15 in a protein complex. To further investigate the effect of ZC3H15 and TRAF2 interaction on NFκB activation, TRAF2 was transiently transfected into SMMC7721-control and -siZC3H15 cells and NFκB reporter gene was detected (Figure 4G). TRAF2 overexpression increased NFκB luciferase activity, knockdown ZC3H15 decreased NFκB activity, and TRAF2 expression rescued the decrease of the transcriptional activity caused by the loss of ZC3H15.

## DISCUSSION

DFRPs increase DRG stability through physical association, possibly by blocking their poly-ubiquitination [13, 17]. DRG1 and DRG2 comprise a highly conserved subfamily of GTP-binding proteins thought to regulate cell growth [18, 19]. In the present study, we demonstrated that ZC3H15, which interacts with DRG1, is highly expressed in HCC and regulates tumor cell proliferation *in vitro* and *in vivo*.

In our cohort of 276 patients, ZC3H15 expression in HCC tumors was significantly associated with high vascular invasion (P=0.0049) and high serum AFP levels (P=0.0064). AFP is a major plasma protein produced by the yolk sac and the liver during fetal development. It is also widely used as a tumor marker for diagnosis and surveillance of HCC. The correlation between ZC3H15 and serum AFP levels, suggests ZC3H15 may be a proto-oncogene that regulates cell differentiation in HCC. Histological grading may reflect cellular differentiation in HCC, which is regarded as a sign of tumor progression and increased malignancy. Although not statistically significant, there was a trend toward increasing tumor ZC3H15 expression from better to poorer grades (Table 1).

The most commonly accepted survival indices are based on tumor staging and histopathologic observation such as tumor size, number of nodules, and vascular invasion [20–22]. To determine its value as a new cancer biomarker that would assist in the follow-up management after surgery, we examined and characterized ZC3H15 as an independent prognostic factor of outcomes of HCC patients. Using multivariate Cox regression analyses, ZC3H15 expression in HCC tumors correlated with a decreased overall survival rate and increased tumor recurrence. Venous infiltration is defined as local resident tumor infiltration through all vessel wall structures, including the endothelial layer, and is an indication of free tumor entry into blood circulation [23–25]. In our cohort, ZC3H15 levels were closely associated with venous invasion. The data presented here suggest ZC3H15-high HCC patients would be more likely to suffer from tumor metastasis and recurrence after curative therapies than the low expressers. Assessment of ZC3H15 expression level in the resected HCC tissue may thus provide a valuable indication for effective follow-up management, especially for those patients with modest tumor staging or histopathologic features.

To validate the clinical results, we studied the biological function of ZC3H15 at the cellular level. Suppression of ZC3H15 inhibited cell proliferation and growth *in vitro* and *in vivo*. Furthermore, ZC3H15 knockdown induced significant apoptosis in HCC cells. When we used a microarray to assess gene expression in wild type and ZC3H15 knockdown SMMC-7721 cells, we found that components of the WNT signaling pathway, EGF pathway, PDGF pathway, NFκB pathway, and TGFβ pathway all showed marked differences between ZC3H15-high and -low cells (Supplementary Table S2). This suggests ZC3H15 profoundly effects cancer cell proliferation and survival in HCC. NFκB luciferase reporter gene assays showing that ZC3H15 knockdown decreased NFκB transcriptional activity in HCC cells corroborated this finding. Capalbo et al. reported that ZC3H15 interacts with the signaling adapter protein tumor necrosis factor receptor associated factor 2 (TRAF-2) in acute myeloid leukemia (AML) [26]. Our results confirm the association between ZC3H15 and TRAF2 in HCC, suggesting that ZC3H15 activates NFκB signaling. TRAF2, a closely related member of the TRAF family, is an important signal transducer for a wide range of TNF receptor superfamily members, including TNFR1 and TNFR2. TRAF2 activates NF-κB as well as MAPK and JNK pathways. As a critical proinflammatory cytokine, tumor necrosis factor-alpha (TNF-α) acts as a master switch establishing the intricate link between hepatitis and HCC. Our results show that absence of ZC3H15 reduces activation of NF-κB signaling induced by expression of TRAF2, indicating that the ZC3H15/TRAF2 complex positively regulates NF-κB. Active NFκB controls various target genes, including chemokines, immune receptors, adhesion molecules, stress response genes, regulators of apoptosis, transcription factors, growth factors, enzymes, and cell-cycle regulators [27, 28]. This finding indicates an important function of ZC3H15 in HCC involves NFκB signaling, though the precise mechanism by which ZC3H15 affects HCC tumorigenesis and progression remains to be determined in future studies.

## MATERIALS AND METHODS

### Patients and clinical samples

We recruited a cohort of 276 consecutive patients with HCC from January 2002 to June 2007 at Eastern Hepatobiliary Surgery Hospital, Second Military Medical University, Shanghai, China. Tumor differentiation was defined according to the Edmondson grading system and tumor staging was determined according to the sixth edition of the tumor-node-metastasis (TNM) classification of the International Union Against Cancer. These selection criteria included patients who underwent surgery alone without chemotherapy or radiotherapy at a time when these adjunctive therapies were not the standard of treatment. All patients included had available paraffin embedded tumor tissues. 15 patients were excluded because of history of other solid tumors. The follow-up period was defined as the interval from the date of operation to the date of death or the last follow-up. Deaths from other causes were treated as censored cases. All patients were observed until June 2012, a period ranging from 1 to 102 mo (median, 22 mo). Overall survival (OS) was defined as the interval between the dates of surgery and death. Disease-free survival (DFS) was defined as the interval between the dates of surgery and recurrence; if recurrence was not diagnosed, patients were censored on the date of death or the last follow-up. Sixty additional pairs of samples from HCC patients who underwent primary hepatectomy in our hospital from January 2010 to December 2011 were selected for PCR and western blot assays. These samples included patients who underwent surgery alone without chemotherapy or radiotherapy and all had available frozen and paraffin embedded tumor tissues. Eighteen normal liver tissues were collected from patients with hemangioma as normal control. Patient samples were obtained following informed consent according to an established protocol approved by the Ethics Committee of Eastern Hepatobiliary Surgery Hospital.

### Tissue microarray and immunohistochemistry

After screening hematoxylin and eosin-stained slides for optimal tumor content, we constructed tissue microarray (TMA) slides (Shanghai Biochip Company, Ltd., Shanghai, China). Two cores were taken from each formalin-fixed, paraffin-embedded HCC sample and normal liver sample by using punch cores that measured 0.8 mm in the greatest dimension from the center of tumor foci. Immunohistochemistry was performed as previously described [29]. The sections were incubated with primary polyclonal antibodies against ZC3H15 (Santa Cruz biotechnology) at 1:50 dilution. The visualization signal was developed with diaminobenzidine and the slides were counterstained in hematoxylin.

Stained sections were evaluated in a blinded manner without prior knowledge of the clinical information using the German immunoreactive score (IRS) as previously described [30]. IRS assigns sub-scores for immunoreactive distribution (0–4) and intensity (0–3), then multiplies them to yield the IRS score. The percent positivity was scored as “0” (<5%), “1” (5–25%), “2” (25–50%), “3” (50–75%), or “4” (>75%). The staining intensity was scored as “0” (no staining), “1” (weakly stained), “2” (moderately stained), or “3” (strongly stained). Cases with discrepancies in IRS score were discussed with other pathologists until consensus was reached.

### Cell culture, chemicals, and plasmids

HCC cell lines were purchased from Shanghai Cell Bank of Chinese Academy of Sciences (Shanghai, China). All cells were cultured in DMEM media supplemented with 10% fetal bovine serum and 1% antibiotic/antimycotic solution (Sigma-Aldrich, St. Louis, MO), and maintained at 37°C in a humidified atmosphere containing 5% CO_2_. The pcDNA3.1A-TRAF2 plasmid was constructed by Genechem biotechnology (Shanghai, China).

#### RNA collection, cDNA synthesis, and real-time PCR analysis

Total RNA was extracted from fresh-frozen tumor specimens, healthy control tissues, and cell lines in Trizol (Invitrogen, Carlsbad, CA). Reverse transcription of total RNA was performed using random hexamers (Roche Diagnostics, Penzberg, Germany) and SuperScriptII reverse transcriptase (Invitrogen). Polymerase chain reaction (PCR) amplifications of the respective genes were carried out with 40 ng complementary DNA, 500 nM forward and reverse primer, and Evagreen (Gentaurer, Brussels, Belgium) on RG-3000 Real Time Thermal Cycler (Corbett. Research, Sydney, Australia) in a final volume of 20 μl. The primers for ZC3H15 were as follows: forward: TCCCATGACTTGACTCTGGAG; reverse: ACCGTGCTTCTTGTTCACTAC.

#### Cell growth assay

Cell growth was measured via multiparametric high-content screening (HCS). Briefly, SMMC7721-Mock, -Control and -siZC3H15 cells in logarithmic phase were digested, resuspended, counted, and inoculated in 96-well plates at 37°C with 5% CO2 for 5 days. Plates were processed with the ArrayScan™ HCS software (Cellomics Inc.) for each day's analysis. The system is a computerized, automated fluorescence-imaging microscope that automatically identifies stained cells and reports the intensity and distribution of fluorescence in each individual cell. Images were acquired for each fluorescence channel, using suitable filters and 20 × objective. Images and data were stored in a Microsoft SQL database for easy retrieval. For clonogenic assays, different cell lines were seeded in 6-well plates (5,000 cells/well) and grown under the indicated conditions for 10 days. The number of colonies (defined as cell clusters consisting of at least 50 cells) was quantified.

### Western blotting

Western blotting was performed as previously described [31]. Tissues or cells were lysed in RIPA buffer and sonicated. Protein concentrations were measured by BCA (Thermo Scientific, 23228). Proteins were loaded on standard SDS–PAGE gels, transferred to polyvinylidene difluoride membranes, and incubated with the primary antibodies, followed by a fluorescently-conjugated secondary antibody (IRDye800CW, 926-32210). The fluorescence density on PVD membranes was measured on a LI-COR imaging system (LI-COR Biosciences, 9201-01).

### Assay of luciferase reporter gene expression

HCC cells were co-transfected with a mixture of luciferase reporter plasmid and pRL-TK plasmids by PEI indicated in the text. Plasmid DNA amounts were equalized via empty control vector. After 24 h, luciferase activities were measured with Dual-Luciferase Reporter Assay System (Promega) according to the manufacturer's instructions.

### Assessment of cell death

Cells were cultured in DMEM and stimulated with phosphate-buffered saline (PBS) or TNFα (20 nM) + CHX for 0-9 h. To assess the level of cleaved PARP, the cells were harvested and analyzed by western blotting. Alternatively, cells were incubated with PI (P4170, Sigma-Aldrich, St. Louis, MO) for 30 min, washed, resuspended in PBS, and analyzed by flow cytometry (MoFlo XDP Cell Sorter, Beckman) and fluorescence microscopy (Leica).

### Xenograft tumor model

Six-week old nude mice were purchased from the Shanghai Experimental Center (CSA, Shanghai, China). All animal experiments met the requirements of the Second Military Medical University Animal Care Facility and the National Institutes of Health guidelines. 5×10^6^ SMMC7721-control and -siZC3H15 cells suspended in 100 ml of Hanks’ buffered saline solution were injected subcutaneously into the flanks of nude mice. Tumor volumes were measured using calipers every 5 days.

### Statistical analysis

The Pearson *x*^2^ test or Fisher's exact test was used to analyze the relationship between ZC3H15 expression and clinicopathologic features. Survival curves were calculated using the Kaplan-Meier method and compared by the log-rank test. The Cox proportional-hazard regression model was used to explore the effect of the clinicopathological variables and ZC3H15 expression on survival. SPSS 15.0 software (SPSS Inc., Chicago, IL, USA) was used for all statistical analyses and a p value < 0.05 was considered significant.

## SUPPLEMENTARY TABLES




