# Supplementary material for: Elevated ZC3H15 increases HCC growth and predicts poor survival after surgical resection
Source: Oncotarget. 2016 May 14;7(24):37238–49. doi: 10.18632/oncotarget.9361 (PMC5095072; doi:10.18632/oncotarget.9361)
Supplement: Supplementary file 1 [file oncotarget-07-37238-s001.pdf]

Elevated ZC3H15 increases HCC growth and predicts poor survival after surgical resection

SUPPLEMENTARY TABLES

Supplementary Table S1: Relationship between DFRP1 protein expression and clinicopathologic characteristics of HCC (n=76*).

See Supplementary File 1

Supplementary Table S2: Identification of signaling pathways influenced by DFRP1 by microarray

Pathway	Gene number	Changed gene	Gene Symbol(FC)	%
Insulin Signaling Pathway	22	2	JUN(-4.490459)	9.09%
Insulin Signaling Pathway	22	2	CSNK2A1(-4.3358665)	9.09%
PDGF Signaling Pathway	29	2	JUN(-4.490459)	6.90%
PDGF Signaling Pathway	29	2	CSNK2A1(-4.3358665)	6.90%
AKT Signaling Pathway	23	1	FASLG(12.407429)	4.35%
NF-kB Signaling Pathway	24	1	TNF(7.363243)	4.17%
EGF Signaling Pathway	28	2	JUN(-4.490459)	7.14%
EGF Signaling Pathway	28	2	CSNK2A1(-4.3358665)	7.14%
WNT Signaling Pathway	26	6	CTBP1(4.3070455)	23.08%
WNT Signaling Pathway	26	6	FRAT1(-28.891495)	23.08%
WNT Signaling Pathway	26	6	CREBBP(-4.432927)	23.08%
WNT Signaling Pathway	26	6	CSNK2A1(-4.3358665)	23.08%
WNT Signaling Pathway	26	6	MYC(-6.8308425)	23.08%
WNT Signaling Pathway	26	6	CCND1(-4.163627)	23.08%
PTEN dependent cell cycle arrest and apoptosis	19	1	FASLG(12.407429)	5.26%
Rho cell motility signaling pathway	33	1	ARHGAP6(-7.478171)	3.03%
Integrin Signaling Pathway	38	1	JUN(-4.490459)	2.63%
Ras Signaling Pathway	24	1	RALBP1(-5.780125)	4.17%
p53 Signaling Pathway	17	2	TP53(-4.1172166)	11.76%
p53 Signaling Pathway	17	2	CCND1(-4.163627)	11.76%
TGF beta signaling pathway	20	3	ZFYVE9(5.640574)	15%
TGF beta signaling pathway	20	3	CREBBP(-4.432927)	15%
TGF beta signaling pathway	20	3	EP300(-4.515618)	15%
