# Supplementary material for: Elevated ZC3H15 increases HCC growth and predicts poor survival after surgical resection
Source: Oncotarget. 2016 May 14;7(24):37238–49. doi: 10.18632/oncotarget.9361 (PMC5095072; doi:10.18632/oncotarget.9361)
Supplement: Supplementary file 2 [file oncotarget-07-37238-s002.docx]

**Table S1 Relationship between DFRP1 protein expression and clinicopathologic characteristics of HCC (n=76***)

| **Characteristics** | **No. patients(%)** | **DFRP1 immunoreactivity** | | **P value** | |
| --- | --- | --- | --- | --- | --- |
|  |  | **High** | **Low** | **Univariate*** | **multivariate***  **OR(95%CI)** |
| Gender |  |  |  |  | **0.027** |
| Male | 68(89.47) | 37 | 25 | 0.1388 | **0.114(0.017-0.780)** |
| Female | 8(10.53) | 2 | 6 |  |  |
| Age (yrs) |  |  |  |  |  |
| <40 | 12(15.79) | 5 | 6 | 0.2236 |  |
| 40-60 | 48(63.16) | 29 | 17 |  |  |
| >60 | 16(21.05) | 5 | 8 |  |  |
| AFP |  |  |  |  | **0.031** |
| ≤20(ng/mL) | 31(41.33) | 12 | 16 | **0.0585** | **4.053(1.134-14.486)** |
| >20 | 44(58.67) | 27 | 14 |  |  |
| HBV |  |  |  |  |  |
| Negative | 9(11.84) | 5 | 3 | 0.9741 |  |
| Positive | 67(88.16) | 34 | 28 |  |  |
| Histological grading |  |  |  |  |  |
| Well | 0(0.00) | 0 | 0 | 0.4452 |  |
| Moderate | 15(19.74) | 6 | 7 |  |  |
| Poorly | 61(80.26) | 33 | 24 |  |  |
| Largest tumor size |  |  |  |  |  |
| ≤3cm | 76(100.00) | 39 | 31 | 1.0000 |  |
| 3-10cm | 0(0.00) | 0 | 0 |  |  |
| >10cm | 0(0.00) | 0 | 0 |  |  |
| Presence of satellite nodules |  |  |  |  |  |
| Negative | 23(30.26) | 10 | 12 | 0.2420 |  |
| Positive | 53(69.74) | 29 | 19 |  |  |
| Vascular invasion |  |  |  |  |  |
| Negative | 44(57.89) | 21 | 20 | 0.80 |  |
| Positive | 32(42.11) | 18 | 11 |  |  |
| HbeAg |  |  |  |  |  |
| Negative | 57(75.00) | 27 | 25 | 0.2778 |  |
| Positive | 19(25.00) | 12 | 6 |  |  |
| Tumorous number |  |  |  |  |  |
| 1 | 62(81.58) | 23 | 33 | 0.2791 |  |
| >=2 | 14(18.42) | 8 | 6 |  |  |
| Child-Pugh Classification |  |  |  |  |  |
| Child-A | 65(87.84) | 28 | 33 | 0.9432 |  |
| Child-B | 9(12.16) | 3 | 5 |  |  |
| UICC stage |  |  |  |  | **0.028** |
| I+II | 68(89.47) | 25 | 37 | 0.1393 | **0.077(0.008-0.755)** |
| III+IV | 8(10.53) | 6 | 2 |  |  |
